# Medical News

**Published:** 1852-06

**Authors:** 


### MEDICAL NEWS.

### AMERICAN MEDICAL ASSOCIATION:

*Abstract of Proceedings of its late meeting held at Richmond, Va., May 4, 1852.*

[From the New Jersey Medical and Surgical Reporter.]

The Association met in the Second Presbyterian Church, at 11 o'clock. The President, Dr. Moultrie, of Charleston, S. C., in the chair.

Dr. James Beale, Chairman of the Committee of Reception, welcomed the delegates to the city of Richmond.

Dr. Haxall, Chairman of Committee of Arrangements, read a list of the delegates who were present, and registered.

Dr. Hays, of Pa., offered the following resolution :—

*Resolved*, That a committee of one from each State, to be selected by its delegation, be appointed to nominate suitable officers for the Association.

The resolution having been adopted, a recess of ten minutes was taken, to allow the delegations opportunity to appoint the Nominating Committee.

At the expiration of the recess the President announced the following committee.

*Nominating Committee.*

| | | | |
|---|---|---|---|
| Isaac Lincoln, | *Maine.* | J. H. Dickson, | *N. C.* |
| Jeremiah Blake, | *N. H.* | H. R. Frost, | *S. C.* |
| Jacob Bigelow, | *Mass.* | C. B. Nottingham, | *Geo.* |
| H. W. Rivers, | *R.I.* | A. Lopez, | *Ala.* |
| Chas. Hooker, | *Conn.* | W. L. Sutton, | *Ky.* |
| Jos. M. Smith, | *N.Y.* | C. A. Pope, | *Mo.* |
| G. R. Chetwood, | *N.J.* | D. Tilden, | *Ohio.* |
| G. W. Norris, | *Penn.* | D. Brainard, | *Ill.* |
| H. T. Askew, | *Del.* | Z. Pitcher, | *Mich.* |
| G. S. Gibson, | *Md.* | J. H. Rauch, | *Iowa.* |
| C. Boyle, | *D. C.* | Paul T. Eve, | *Tenn.* |
| James Beale, | *Va.* | | |

After the calling of the Roll the President delivered the Annual Address.

The Nominating Committee then reported the following gentlemen officers of the Association for the present year.

*For President*—Beverlly R. Welford, M. D. of Va.

*For Vice Presidents*—Jonathan Knight, M. D,. of Conn., James W. Thomson, M. D., of Del, Thos. S. Simons, M. D., of S. C., Chas. A. Pope, M. D., of Missouri.

*For Treasurer*—D. Francis Condie, M. D. of Pa.

The officers thus nominated were duly elected, and the committee requested to nominate Secretaries, and to decide upon the next place of meeting.

On motion, Drs. John L. Atlee, of Pa., Haxall, of Va., and Eve of Tenn., were appointed a committee to wait upon Dr. Wellford, and announce his election, and conduct him to the chair. Dr. Wellford having taken the chair returned his thanks for the honor conferred upon him.

Dr. Haxall, of Va., offered the following preamble and resolution, which were unanimously adopted :—

"The American Medical Society in Paris being so constituted, that it would be entitled to representation if it existed in this country, and as it is recognized abroad as an American institution ;

"*Resolved*, That the delegates accredited to the Association by the American Medical Society in Paris, be, and are hereby invited to take seats in this body."

Dr. Paul Lajus offered the following resolution, which was adopted :

*Resolved*, That Dr. Brown-Sequard of Paris be invited to occupy a seat among the delegates at the present meeting of the Association.

Dr. Isaac Hays read the Report of the Committee on Publication, and the Report of the Treasurer. They were received, and the following resolutions appended to the report of the Committee of Publication were unanimously adopted.

*Resolved*, That the assessment for the present year be three dollars.

*Resolved*, That the Committee of Publication be authorized to fix the price at which the Transactions for the present year will be furnished to such of the members of the Association as shall remit the amount decided upon by the committee within a specified time (to be fixed by them.) And that it shall be the duty of the said committee, to issue a circular, informing the members of the terms upon which the transactions will be furnished them.

*Resolved*, That the Committee be further authorized to take such measures in the disposal of the copies of the Transactions, remaining after all such members are supplied as shall comply with the terms set forth in the circular of the Committee, as they may deem expedient.

Dr. Hayward presented the report from the Committee on Prize Essays, and broke the seal of the packet, containing the name of the author of the essay entitled, "*On Variations of Pitch in Percussion and Respiratory Sounds and their application to Physical Diagnosis*," which was deemed worthy of the prize. The author proved to be Dr. Austin Flint, of Buffalo, N. Y., to whom the prize was awarded, and the report referred to the Committee of Publication.

The Report of the Committee on the Medical Botany of the U. S. for 1850-51 from Dr. A. Clapp, Conn., was presented and referred to the Committee of Publication.

Dr. R. W. Haxall, of Va., read a short report of the progress of the Committee on the Epidemics of Virginia and North Carolina, and asked to be continued ; which request was granted.

On motion of Dr. Gooch, the Editorial Corps were invited to take seats on the floor.

The Secretary stated that he had transmitted copies of the preamble and resolutions of 1850–51, relative to assimilated rank of the medical staff of the army and navy, to the several departments ordered by the resolutions, and that the chief of the Bureau of Medicine and Surgery at Washington, had approved the course recommended by the Association in a letter which was read. Dr. Pinkney, of the Navy, asked leave to read a memorial, which he had prepared to present to Congress on this subject, and which he sustained in a lengthened and forcible speech—after which Dr. Cox, of Md., offered resolutions expressive of the approval of the Association in the matter, &c. &c.—which resolutions were referred to a special committee.

Dr. Simons, of S. C., offered the following resolutions:

*Resolved*, That the American Medical Association do memorialize Congress to require all vessels bringing steerage passengers over to have a surgeon on board.

*Resolved*, That a committee of this Association be appointed to draw up a memorial to Congress, making such suggestions as they may deem fit as regards the importance of this measure.

The report of the Committee upon the Constitution, being the special order—Dr. Hays, chairman of the Committee, made a Report. Dr. T. . Yardley, a member of the committee, presented a counter-report. Much discussion ensued, and many resolutions and amendments were proposed, and withdrawn in favor of the following resolution, offered by Dr. Thomas, of Md., and amended by Dr. Stewart of N. Y.

*Resolved*, That the two Reports, on proposed alterations of the Constitution, be referred to a committee of three, to be appointed by the chair, with instructions to report to-morrow morning in definite and proper form, such amendments as will embrace the views set forth in the reports, and such other views as may appear to them advisable.

The resolution was adopted.

The Secretary read the following communication from the New York Academy of Medicine, which on motion was referred to the publishing committee, and ordered to be printed.

New York Academy of Medicine,
New York, *April*, 22, 1851.

Sir: I have the honor to transmit to you a copy of the preamble and resolutions adopted at a regular meeting of the New York Academy of Medicine, held April 21, 1852.

*Whereas*, The clinics, now held at the medical colleges, as at present conducted, are, or may be made tributary to the private interests of the professors, at the expense of other and younger members of the profession—depriving them, by an odious monopoly, of practice and operations, and often of *fees* to which they are justly entitled,

*Therefore, Resolved*, As the sense of this Academy, that to prescribe or practice upon the legitimate patients of any other physician, knowing them to be such, although done gratuitously at a clinic, is equally unwarrantable and unprofessional, with similar interference with the patients of another in private practice, and in either case is a violation of the code of medical ethics, adopted by this body.

*Resolved*, That the possible perversion of these clinics, to the private emolument of those conducting them, by transferring patients to their private offices, and then exacting fees from those found able to pay, divests the clinics of all pretext for professing to be public charities, and should be scrupulously guarded against in all our colleges by stringent rules.

*Resolved,* That a copy of these resolutions be sent to the authorities of the several medical colleges in this city.

The Secretary was also instructed to forward a copy of these resolutions to the American Medical Association.

Respectfully yours,
JACKSON BALTON, M. D., *Recording Secretary.*

Dr. Eve, from the committee on nominations, recommended the following additional officers for the ensuing year:—

*For Secretaries*—Drs. P. Claiborne Gooch, of Va., and Edw. L. Beadle, of N. Y.

*Committee of Publication*—Drs. D. F. Condie, of Pa., Isaac Hays, of Pa., P. C. Gooch, of Va., E. L. Beadle, of N. Y., Isaac Parrish, of Pa., G. Emerson, of Pa., G. W. Norris, of Pa.

*Committee of Arrangements*—Drs. F. Campbell Stewart, of N. Y., John Watson, of N. Y., Wm. Rockwell of N. Y., James R. Wood, of N. Y., Robt. Watts, Jr., N. Y., Alfred Post, of N. Y., John G. Adams, of N. Y., H. D. Bulkley, of N. Y.,

On motion, the Report was received, and the gentlemen named were unanimously elected officers of the Association.

The Report of the Committee on "The Blending and Conversion of the Types of Fever," was next read by Dr. C. B. Williman, S. C. (in place of Dr. Dickson, not present,) and referred to the Committee of Publication.

Dr. Hayward, of Mass., presented and read a Report upon the Radical Cure of Hernia, which was also referred to the Publishing Committee.

Dr. Greene, N. Y., offered the following resolutions:—

*Resolved,* That at all future meetings of this Association, the reports of Committees and all contributions on scientific subjects, occupying more than ten pages of quarto post manuscript, be accompanied each by an abstract or synopsis embracing the principal points of such report or paper, which abstract or synopsis may be read before the Association.

*Resolved,* That the above resolution be transmitted to the Chairman of each Committee.

The thanks of the Association were presented to the physicians and citizens of Virginia, to the Trustees of the Second Presbyterian Church, to the Committee of Arrangements, and to the Managers of the Danville Railroad Company, for their kindness and attention to its members.

The following committee was appointed to report what action this Association should take in order to procure, for distribution among the medical profession of the United States, a large edition of the medical statistics of the country, as furnished by the late census, viz.: Drs. Simons, of S. C., S. Boyle, of D. C., Summer, of Conn.

The following Committee was appointed to report on the relation between climate and pulmonary consumption, viz.: Drs. D. F. Condie, R. E. Rodgers, James M. Smith, Moultrie, and McGuire.

On motion of Dr. Rockwell, it was *Resolved*, That the Committee to memorialize Congress on the subject of compelling passenger vessels to carry surgeons, be directed also to call their attention to the importance of giving to each steerage passenger a certain amount of space between decks.

The Nominating Committee reported the following special committees:—

D. F. Condie, Philadelphia, Causes of Tubercular Disease.

James Jones, New Orleans, The Mutual Relations of Yellow and Bilious Remittent fevers.

R. S. Homes, St. Louis, Missouri, Epidemic Erysipelas.

Charles D. Meigs, Philadelphia, Acute and Chronic Diseases of the Neck of the Uterus.

J. P. Jervey, Charleston, South Carolina, Dengue.

Daniel Drake, Cincinnati, Milk sickness, so called.

Dr. Lopez, Mobile, Prevalence of Idiopathic Tetanus.

George B. Wood, Philadelphia, Diseases of Parasitic Origin.

R. D. Arnold, Savannah, the Physiological Peculiarities and Diseases of Negroes.

Joseph Carson, Philadelphia, Alkaloids which may be substituted for Quinia.

S. D. Gross, Louisville, Ky., Results of Surgical Operations for the relief of Malignant Diseases.

James R. Wood, New York, Statistics of the Operation for the Removal of Stone in the Bladder.

Alexander H. Stevens, New York, Sanitary Principles applicable to the Construction of Dwellings.

G. Emerson, Philadelphia, Agency for the Refrigeration produced through Upward Radiation of Heat, as an Exciting Cause of Disease.

Henry J. Bigelow, Boston, The best Means of Making Pressure in Reducible Hernia.

A. T. B. Merrit, Richmond, Cholera and its Relations to Congestive Fever; their Analogy or Identity.

Ushur Parsons, Providence, R. I., Displacement of the Uterus.

H. F. Campbell, Agusta, Ga., Typhoid Fever.

Worthington Hooker, Connecticut, Epidemics of New England and New York.

Jno. L. Atlee, Lancaster, Pa., Epidemics of New Jersey, Pennsylvania, Delaware, and Maryland.

R. W. Haxall, Richmond, Va., Epidemics of Virginia and North Carolina.

William M. Bolling, Montgomery, Ala., Epidemics of South Carolina, Georgia, Florida, and Alabama.

Edward H. Barton, New Orleans, Epidemics of Mississippi, Louisiana, Texas, and Arkansas.

Dr. Sutton, Georgetown, Ky., Epidemics of Tennessee and Kentucky.

Thomas Reyburn, St. Louis, Mo., Epidemics of Missouri, Illinois, Iowa, and Wisconsin.

George Mendenhall, Cincinnati, Ohio, Epidemics of Ohio, Indiana and Michigan.

*Committee on Volunteer Communications.*

| | |
|---|---|
| Joseph M. Smith,<br>Jno. A. Swett,<br>Willlard Parker,<br>Gurdon Buck,<br>Alfred C. Post, | all of New York. |

The titles of the Reports on Epidemics and other special reports were read, and the Reports referred to the Committee on Publication.

The subject of altering several important provisions of the constitution presented in a majority and minority report, and both submitted by different portions of a committee, to whom the subject was referred last year, claimed a considerable share of attention in the Association, and much interesting discussion ensued in which some of the most distinguished men of our profession participated; and the Committe who were charged with condensing the subjects of both reports into one, so far as they could be made compatible with each other, presented a series of well-digested propositions, which, after free discussion and some alterations, were laid on the table till next year, then to be taken up and finally acted upon. These propositions embrace the absorbing question of delegation, and present a conservative policy, which may probably be adopted, as it is evident the Association is not yet fully prepared to exclude the schools from a representation at its meetings.

The Committee suggest that delegates shall be appointed for one year, that they shall hold their appointment from County and State Medical Societies, from chartered medical colleges, hospitals, and permanent voluntary medical associations in good standing with the profession, and from the medical staffs of the army and navy. The county, district, and voluntary chartered medical societies, shall have the privilege of sending one delegate for every ten of its resident members, and one more for every additional fraction of more than one-half that number; while every State Society may send four delegates, and, in those States in which county and district societies are not generally organized, they shall be entitled to send one for every ten regular members, and one more for every additional fraction of more than half of this number. No medical society shall have the privilege of representation which does not require of its members an observance of the code of ethios adopted by the Association. Every chartered medical college, acknowledging fealty to the code of ethics, having six professors, and giving a course of lectures of not less than sixteen weeks annually, requiring also of students three years study; and hospitals, and lunatic asylums, having accommodations for one hundred patients, shall be entitled each to one delegate. The University of Virginia, in consequence of its peculiar mode of instruction and examination, was mentioned as worthy of representation, notwithstanding it does not comply, in some respects, with the terms of the proposition; but the exception in its favor is only to be continued so long as the present system of teaching shall remain in force.

### PROCEEDINGS OF THE MEDICAL SOCIETY OF THE STATE OF PENNSYLVANIA,

*At its Annual Session, Philadelphia, May,* 1852.

PHILADELPHIA, MAY 26th, 1852.

The Medical Society of the State of Pennsylvania convened, pursuant to adjournment from the last annual session, in the Hall N. W. corner of Chestnut and Twelfth streets, and was called to order, at half-past eleven o'clock, A. M., by the President, CHARLES INNES, M. D., of Northampton county. On motion of Dr. WEST of Philadelphia, the President appointed a committee, consisting of Drs. WEST, Shannon, of Schuylkill, and Townsend, of Chester, to examine and verify the credentials of delegates. The committee reported the following :

[Those to whose names an asterisk is appended were in attendance during the session.]

*Berks.*—William Gries, M. D.,* Edward Wallace, M. D.,* D. L. Beaver, M. D.,* Caleb Leggett, M. D.,* P. G. Bertolet, M. D.,*

*Blair.*—J. M. Confer, M. D.,* John D. Ross, M. D.,*

*Bucks.*—William S. Hendrie, M. D., O. P. James, M. D.,* Ralph Lee, M. D., Samuel Carey, M. D., James S. Rich, M. D.,* Charles Foulke, M. D.

*Chester.*—Wilmer Worthington, M. D., Andrews Murphy, M. D.,* Joseph Hickman, M. D.,* John P. Edge, M. D.,* Septimus A. Ogier, M. D.,* A. K. Gaston, M. D.,* W. W. Townsend, M. D.,* Isaac R. Walker, M. D.,* Samuel H. Harry, M. D.,* Bartholemew Fussell, M. D.

*Delaware.*—Samuel A. Barton, M. D., M. Emanuel, M. D.,* R. K. Smith, M. D., * George Martin, M. D.*

*Erie.*—C. F. Perkins, M. D.,* F. W. Miller, M. D.,* Jacob Vosburg, M. D., W. B. Williams, M. D., B. E Phelps, M. D.

*Huntingdon.*—J. G. Lightner, M. D.,* Jonathan H. Dorsey, M.D.,* Mordecai Massey, M. D., J. B. Luden, M. D.*

*Lancaster.*—F. S. Burrowes, M. D., F. A. Muhlenberg, M. D., John L. Atlee, M. D., Samuel Duffield, M. D., J. B. Stubbs, M. D., J. L. Ziegler, M. D., Joseph Gibbons, M. D., J. A. Ehler, M. D.,* Henry Carpenter, M. D., P. Cassidy, M. D.,* C. O. Richards, M. D., E. L. Baer, M. D., Thomas Ellmaker, M. D.

*Lebanon.*—J. Breitenbach, M. D.

*Mercer.*—John T. Ray, M. D.*

*Mifflin.*—George V. Mitchell, M. D.,* Thomas Van Valzeh, M. D.*

*Montgomery.*—Hiram Corson, M. D.,* H. Geiger, M. D.,* Wm. A. Van Buskirk, M. D.,* G. W. Wimley, M. D.*

*Northampton.*—Charles Innes, M. D.*

*Perry.*—A. C. Stees, M. D.*

*Philadelphia.*—G. B. Wood, M. D.,* Samuel Jackson, M. D., 8th street,* Joseph Pancoast, M. D., T. D. Mütter, M. D.,* H. S. Patterson, M. D.,* I. Parrish, M. D.,* Samuel Jackson, M. D., Spruce st.,* Wm. Pepper, M. D.,* I. Hays, M. D.,* George Fox, M. D.,* G. Emerson, M. D.,* A. Stillé, M. D.,* G. W. Norris, M. D.,* H. Bond, M.D.,* F. West, M. D.,* P. B. Goddard, M. D.,* T. F. Betton, M.D.,* E. F. Leake, M. D.,* A. Naudain, M. D., Ed. Janvier, M. D., J. M. Pugh, M. D.,* I. Remington, M. D.,* Jos. Warrington, M. D.,* G. W. Patterson, M. D.,* Wm. Mayburry, M. D.,* T. H. Yardley, M. D.,* D. F. Condie, M. D.,* Wm. Henry, M. D.*

*Schuylkill.*—Samuel H. Shannon, M. D.,* James S. Carpenter, M. D.,* David I. McKibbin, M. D.,* George Halberstadt, M. D.,* J. M. Koehler, M. D.

The Secretary, Isaac Remington, M. D., called the roll, and 53 delegates answered to their names.

Drs. E. W. Hale, of Mifflin, and C. W. Parish, of Chester, were admitted to seats by resolution.

On motion of Dr. Fox, the reading of the minutes of the last session was dispensed with.

On motion of Dr. Shannon, the President read the annual address, prepared in accordance with the resolution of the last session.

This address was exceedingly eloquent and instructive, and was listened to with great interest and satisfaction by the Society. Dr. Innes adverted to the origin and present condition of the Society, and, while he congratulated the members upon what had been accomplished in the cause of progress, he could not admit that the survey was altogether as flattering as might be desired. It was certainly a matter of some reproach that the movement for a general organization of the profession in Pennsylvania had been so long deferred. Stimulated by the example of sister States, and at the immediate solicitation of the medical societies of the counties of Chester and Lancaster, the State Medical Society was organized in the year 1848—a movement which he felt he might safely say was not premature. This was the fifth annual session of the Society. The assemblage before him was certainly a very gratifying representation of the profession of the State, but he could not withhold the expression of his regret that so much territory within its borders remained unrepresented. Scarcely one third of the State had been represented at the last session, and but 17 of our 63 counties have formed auxiliary local associations. This was indeed lamentable apathy, and a far less cordial response of the profession than might have been expected. Whence, he asked, this reluctance to organize county societies? and why did so many of our brethren refuse to come into fellowship with us? He was indeed at a loss to understand this non-appreciation of the social and professional advantages to be derived from a commingling of the members of the profession. As regards the hostility to the county Societies, which had been elicited in some quarters, he must avow his frank conviction that it could proceed only from an absence of true professional spirit, and from an indisposition to look beyond individual aggrandisement. Indeed he must assign to selfish and mercenary motives the active opposition which had been advanced to the late attempts to organize the profession in our State, and he trusted that all who had remained aloof, from mere indifference, would not for the future abstain from lending their names and influence to the cause of association.

Dr. Innes commented with deserved severity upon the sinister and sordid views which had induced many to abstain from uniting in this organization. He felt that a disinclination to adhere to a proper fee bill was a motive, with at least some, for standing off; and he handled with just and pointed sarcasm the "cheap doctors," who underbid their compeers, to obtain practice, and brought our noble profession down to the level of a mere trade. He cited, as worthy of imitation, the example of the Western Medical Society of Wisconsin, which obliged the Corresponding Secretary to invite all practitioners residing in the State to become members of the State Society, with a proviso that those who should fail to respond to the appeal, within six months, should be deemed irregular, and be cut off from professional intercourse. The Medical Society of Northampton county, Pennsylvania, had resolved to consult only with members of their own or other county societies. In Erie county, also, the members of the county Society had adopted the same course, and declined all professional recognition of those who remained without the pale of their organization. These measures might seem ultra, but they were called for by the exigencies of the case, and he urged a non-intercourse policy—the argumentum ad hominem—as the only means of reaching the recusants. Some other lever than moral suasion was certainly needed to move them.

In conclusion, Dr. Innes expressed his approval of the recommendations of the Philadelphia County Medical Society, as regards State representation in the National Medical Association.

On motion of Dr. Gries, of Berks, the thanks of the Society were unanimously tendered to the President for the Address just delivered, and it was ordered to be published in the Minutes.

On motion of Dr. Condie, of Philadelphia, the President appointed the following Committee to audit the Treasurer's accounts: Drs. Condie, Ehler, of Lancaster, and Ross, of Blair.

On motion of Dr. Hays, of Philadelphia, it was ordered that the President appoint a Committee of one delegate from each County to nominate Officers for the ensuing year.

Dr. Hays, as Corresponding Secretary, announced that since last year, but one additional county, *Cambria,* had formed a County Medical Society.

The Auditing Committee now reported that they had examined the Treasurer's accounts, and found a balance in hand of $123.41. $150 had been received from the delegates during the past year, and $26.59 had been drawn out. The report was accepted and the Committee discharged.

Dr. Condie, from the Committee on Publication, reported that the Proceedings of the last session had been published and duly distributed in September last; and that the County Societies, with three exceptions, had forwarded the sums assessed upon them for the expenses of publication. He thought it proper to mention that the counties which had not yet forwarded their quotas, were Mifflin, York, and Lycoming.

Dr. VAN VALZEH, of Mifflin, stated that the amount due from Mifflin had been collected, and if it had not been forwarded, it was doubtless through want of opportunity.

Dr. EHLER, of Lancaster, moved that the balance remaining in the hands of the Committee on Publication, be paid over to the Treasurer.

Dr. HAYS suggested to Dr. Ehler to amend his resolution so as to authorize the Committee on Publication to retain the balance now in their hands.

But, after a brief debate, the resolution of Dr. Ehler was adopted in its original form.

On motion of Dr. CONDIE, it was resolved that no assessment be made upon the delegates for the present session.

The following resolutions were, on motion of Dr. CONDIE, adopted on the subject of Publication : 1st, the resolution passed at the last session, in reference to printing the Proceedings, was re-enacted in the same form; 2d, the chairman of the Committee on Publication was directed to pay over to the Treasurer all funds hereafter received from county societies, to be subject to the order of the Committee on Publication, together with the remaining balance in the Treasurer's hands, for the expenses of publication.

Dr. FOX, of Philadelphia, announced that he declined being considered a candidate for re-election to the office of Treasurer.

The Society now adjourned to half past four, P. M.

### AFTERNOON SESSION.

The Society met, pursuant to adjournment, the President in the chair.

The President announced, as the Committee to nominate officers,—Drs. Hays, of Philadelphia, James, of Bucks, Gries, of Berks, Confer, of Blair, Murphy, of Chester, Martin, of Delaware, Perkins, of Erie, Dorsey, of Huntingdon, Van Buskirk, of Montgomery, Cassidy, of Lancaster, Van Valzeh, of Mifflin, Stees, of Perry, Shannon, of Schuylkill, Breitenbach, of Lebanon, and Ray, of Mercer.

The reports from the county Medical Societies were called for, and and were submitted and read seriatim. They were exclusively from the interior counties,—Philadelphia county, which presented so able a report last year, having this year been derelict. The reports were received by the Society with great satisfaction, and elicited the warmest commendation in all quarters, for the spirit, research and general ability by which they were characterized. It was felt by all who had the good fortune to listen to these interesting and instructive papers, that the Society had already accomplished much, in developing so large an amount of valuable statistical information on the medical topography and diseases of our State.

The counties from which reports were presented were, Schuylkill, Delaware, Mifflin, Chester, Lebanon, Blair, Bucks, Huntingdon, Berks, Erie, Lancaster, and Perry. The reports from the three last-named counties were limited to notices of the proceedings, and lists of the officers and members of the county societies. A report, not presented, was prepared from Montgomery, which is to be handed to the Committee of Publication.

Dr. Halberstadt, of Schuylkill, presented and read the report from that county, appended to which was a beautifully executed geological chart of the county. This report, which was drawn up with admirable conciseness, contained a brief sketch of the organization of the county Medical Society, (now in existence seven years,) a statistical and topographical sketch of the county, with particular reference to the relation between its diseases and geological formations, and a history of the diseases which prevailed during the past year, with the treatment pursued, &c. The report recommended the Society to group together the same geological strata, in distributing the State among Committees for future reports, as the best means of accumulating general results. This suggestion, it will be seen by reference to the later proceedings, the Society adopted.

Dr. Smith, of Delaware, presented and read the report from that county, which was also accompanied by a geological chart. This report contained a preliminary topographical and botanical notice of the county, with meteorological observations for the last four years; followed by a highly interesting account of the diseases observed in the county during the past year. The report expressed a disbelief in the opinion advanced in various quarters, that geological formations influence the development of disease, with the single exception of the prevalence of remittent and intermittent fevers in alluvial districts.

Dr. Condie, (by request), read the report from Mifflin county, which was understood to have been drawn up by Dr. Henderson, of Mifflin, who was not present. This report, the first from Mifflin county, presented a detailed description of the topography and geological formations of the county, designed by the county society, to serve as a *primary* report, which should be the basis of future reports. This was followed by a very interesting sketch of the diseases prevalent in the county, from which we select one or two points which particularly arrested our attention. Diseases of malarious origin are of rare occurrence in the county. Epidemic dysentery is sometimes witnessed; and the exemptions from its visitations, claimed for the *limestone districts*, in the reports of Berks and other counties, has not been noticed in Mifflin. A circumscribed epidemic typhus fever prevailed some time since, which appeared to have been generated originally by decayed matter in a house overflowed with water, and which in its progress manifested the clearest characters of contagion.

The President presented the resignation by Dr. C. D. Gloninger, of the office of recording secretary, which, on motion, was accepted.

The Secretary presented and read the report from Chester county, which was chiefly taken up with descriptions of the diseases prevalent in various sections of the county, by the physicians of the respective localities. Although the committee expressed regret that they had not obtained a more general response from the physicians of the county, they were enabled to present numerous highly interesting details of the medical topography of this large county, which contains, according to the report, one hundred regular physicians. With their next annual report, the Chester county society expects to furnish a geological map of the county.

Before the conclusion of this report, on motion of Dr. Gries, the Society adjourned to half past ten o'clock, A. M., the next day.

*Second day's session, 27th of May,* 1852.

The Society met pursuant to adjournment, the President in the chair.

The reading of the Chester county report was resumed and concluded.

Dr. C. W. Parish, of Chester, moved that the reading of the remaining reports be dispensed with. Dr. Stillé, of Philadelphia, moved as an amendment, that hereafter when reports of committees shall require more than fifteen minutes to be read, the reporters shall present and read abstracts of their contents. Dr. Parish accepted this in lieu of his own resolution.

A brief debate ensued, in which Drs. Stillé, C. W. Parish, and Gries advocated the reading of condensed abstracts of the reports of the county societies, rather than the reports at length; and Drs. I. Parrish, Wood, Emerson, and Hays, deprecated any attempt to cut them short. Dr. I. Parrish urged, that to listen to these reports was the chief purpose for which they had assembled, and he thought the time of the Society would be much better employed in this way, than in protracted discussions upon minor points of business.

On motion of Dr. Hays, Dr. C. W. Parish's motion was laid on the table.

Dr. Hays moved that a committee be appointed to devise a plan for the appointment of committees to collect accounts of epidemics, as they prevail through the different geological formations of the State.

At the suggestion of Dr. Emerson, of Philadelphia, the mover laid this resolution on the table, to be taken up hereafter.

Dr. Harry, of Chester, presented an Appendix to the Chester county report, which, on motion of Dr. Edge, of Chester, was referred, along with the original report, to the Committee on Publication.

Dr. H. S. Patterson, (by request), in the absence of Dr. Gloninger the reporter, presented and read the report from Lebanon county. It offered an interesting sketch of the medical topography of the county, and was accompanied by a well executed map.

The Secretary presented and read the report from Blair county, which was mainly devoted to a description of the prevailing diseases of the county, and the mode of treatment. The report asked the attention of the State Society to the culpable neglect of vaccination which prevails in many of the rural districts.

Dr. Rich, of Bucks, presented and read the report from that county. He stated that there had been no regularly constituted committee of the Bucks County Society, to prepare a report; but that the delegates had been charged with the duty. It had fallen to Dr. Rich's lot, though not the first named among the delegates, to prepare the report, and as he had had but a short notice, and had received no information from the physicians of the county, he could not offer much. Bucks county, however, hoped to do better in future. Notwithstanding the limited resources at Dr. Rich's disposal, he furnished many interesting details of the topography, geology, and diseases of the county. He expressed as the result of his observation, the opinion that the diseases of the county had depended essentially upon the geological formations.

The Secretary presented and read the report from Erie county, which was limited to a report of the proceedings of the county society. A complete report of the medical topography of that county is promised next year.

Dr. Luden, of Huntingdon, read the report from that county. It gave the topography and geological features of the county, but expressed difficulty in deciding, in so mountainous a region as Huntingdon county, how far the geological formations influence the development of diseases. Atmospheric vicissitudes, combined with malaria, appear to be the main causes of disease in that region. The whole valley of the Juniata is a malarious district.

The Report of the Berks County Society was now presented; but, as it was offered in a printed form, in two numbers of the Peoples' Advocate newspaper, of that county, it was objected to as irregular. After some debate it was referred to a special committee, to report at the afternoon session. Drs. H. S. Patterson, I. Parrish, and West, were appointed this committee.

The Secretary presented reports from the Lancaster and Perry County Societies, which were confined to lists of the officers and members of the Societies of these counties.

Dr. Ehler offered a resolution, expressing the regret of the Society at the death of Dr. George B. Kerfoot, of Lancaster, a member of the Society from its origin, and for two years its Recording Secretary. This was unanimously adopted, and the Secretary was directed to forward a copy of the resolution to the family of the deceased.

On motion of Dr. Ray, of Mercer, a similar resolution was adopted in reference to the late Dr. John Baskin, of Mercer, one of the Vice Presidents of the Society.

Dr. Hays offered the following resolution: "That the members of this Society, when associated for educational or other purposes, owe the same fealty to the code of ethics of the Society in their corporate and associate capacities, as they do as individuals." This resolution, Dr. Hays averred, was designed to express disapproval of the puffing strain indulged in by the Faculties of certain Medical Colleges in their Annual Circulars or Announcements.

Dr. Jackson, of Philadelphia, (of the University of Pennsylvania,) had no objection to the resolution, as an abstract proposition; but he was not aware that the practice of any of the Schools was such as had been stated. Nothing of the kind had come under his notice.

After a few remarks from Drs. Wood, Jackson, (late of Northumberland,) and Gries, the resolution was adopted.

Dr. Condie offered the following resolution: "That this Society holds all professional intercourse with irregular practitioners, of whatever name or pretensions, and all countenance of their peculiar views or mode of treatment, as a breach of professional ethics; a persistence in which should preclude the individual so offending from membership in any county Society, or, if he be already a member, should subject him to expulsion."

This resolution was briefly debated by Drs. Gries, Condie, Corson, Betton, Perkins, and H. S. Patterson. Dr. Patterson objected to re-enacting by simple resolution what was already a part of the organic law of the Society. If cases in violation of the principle of the resolution had occurred, other methods of reaching the offenders might be found. They should be brought before their respective County Societies, where they would be more speedily and efficiently dealt with. Dr. Patterson pointed out the authority granted by the constitution to the county Societies to punish delinquent members, and he also cited the article on Consultations from the Code of Ethics as covering the ground.

Dr. Condie now expressed himself satisfied that the subject was provided for in the quarter pointed out by Dr. Patterson, and thereupon withdrew his resolution.

The Society adjourned to meet at half-past four, P. M.

AFTERNOON SESSION.

The Society met, pursuant to adjournment, at half past four o'clock, P. M., the President in the Chair.

Dr. H. S. Patterson, from the committee to whom the Berks County report had been referred, reported: That the Committee deemed the publication in a newspaper irregular, but that it appeared to have been done merely for the convenience of the Society—Dr. J. L. Stewart, the writer of the report, being the Editor of the newspaper in question—and that no disrespect was intended to the State Society. Under these circumstances, the error being clearly from inadvertence, and the paper appearing to possess considerable value, the Committee recommended that it be received and read, and also that the Berks County Medical Society be admonished of the impropriety of the course pursued by them.

The report was adopted, and the report from Berks County was accordingly read by Dr. Wallace, of Berks, and was listened to with much interest and gratification by the Society.

After the report had been read, Dr. Wallace moved that a paper which had been read before the Berks County Medical Society, on the Epidemics of the County, be now read and added to the report of the Berks County Society as an Appendix. The motion was adopted, and Dr. Gries read an interesting paper, in which, after presenting a sketch of the diseases lately prevailing in Reading and its vicinity, he gave a lengthy account of a recent epidemic of *Catarrho-Nervous Fever*, which he had treated successfully with large doses of the sulphate of quinine.

Dr. Wallace presented and read a report from the Berks County Society, on the subject of spurious drugs, appended to which was a series of resolutions, which were afterwards withdrawn by the Berks county delegation.

The reports from the County Medical Societies were, on motion, severally referred to the Committee on Publication.

The President presented the resignation by Dr. REMINGTON of the office of Recording Secretary; which, on motion, was accepted, to take effect from the close of the present session.

On motion of Dr. WIMLEY, of Montgomery, the Committee on Publication was authorized to receive the Report of the Montgomery County Society, if handed in before the publication of the Proceedings of the present session.

Dr. WIMLEY, in accordance with instructions from the Montgomery County Medical Society, offered the following preamble and resolutions:

*Whereas*, At the last meeting of the American Medical Association, the subject of amending the Constitution, so as to change the present mode of representation, was presented by a committee, and will be acted on at the next meeting of the Association, therefore,

*Resolved*, That the State Medical Society of Pennsylvania favors the principle of representation by delegates from *County*, *District* and *State* Medical Societies, as that most accordant with the genius of our institutions, and most likely to promote the permanency and success of the National Association.

*Resolved*, That the admission of delegates from Medical Colleges, Hospitals, and other Medical Institutions, although adapted to the forming stage of the Association, is no longer necessary; and is calculated to produce dissatisfaction amongst the mass of the profession, by creating privileged classes, and by giving an undue influence to physicians in large cities, over those residing in country districts and in the smaller towns.

*Resolved*, That restricting representation to *County*, *District*, or *State* Societies, and conferring upon the members of these bodies the right of membership in the National Association, will, in the opinion of this Society, offer a powerful inducement for Physicians throughout the United States to organize in their respective districts, and thus become constituent branches of the Association, and entitled to all the rights and privileges which such membership can confer.

*Resolved*, That the delegates from this Society be requested to lay these resolutions before the National Association, signed by the officers of the State Society, and that they use their exertions to have the principle of representation herein approved, adopted by that body.

Without taking any action on these resolutions, the Society adjourned to Friday, 28th, at 11 o'clock, A. M.

*Third day's Session, 28th May,* 1852.

Pursuant to adjournment, the Society met at eleven o'clock, A. M., the President in the chair.

On motion of Dr. WIMLEY, the Society proceeded to the consideration of the Montgomery county resolutions on representation in the National Medical Association.

Dr. GRIES took the floor, in support of the resolutions, and read a written speech, of some length, in which he animadverted on the unnecessary multiplication of medical schools witnessed within the last few years, and on the almost total absence of a proper standard of examinations for degrees. He referred, in terms of warm commendation, to the admirable address of Dr. Jackson, (late of Northumberland,) delivered before the Philadelphia County Medical Society in February last, and expressed the hope that the State Society would endorse the principles urged by Dr. Jackson.

On motion of Dr. SMITH, of Delaware, it was now *Resolved*, That the members of the Society be not allowed to speak more than twice, or longer than ten minutes on the same question, without permission.

It was ordered that the Montgomery County resolutions be taken up separately.

Dr. HAYS moved to amend the first resolution, by inserting,—after the words "County, District, and State Medical Societies,"—"and a restricted representation from Medical Colleges, subject to certain conditions."

Dr. HAYS believed that the introduction of these resolutions was an attempt to manufacture public opinion on the subject, and to prejudge the question in advance of the action of the National Medical Association. Under existing circumstances, he admitted that it was inexpedient to continue, unchanged, the present plan of representation in the Association. It was certainly unequal. But, in endeavoring to remedy this inequality, we should not go too far, lest, in the end, we overreached ourselves. If we exclude the Colleges entirely from representation in the Association, they become our masters. Allow them a restricted representation—he was not for extending it beyond a single representative—and we control them. By thrusting *all* the schools out of the Association, the good and the bad together, we shall be really giving countenance to those whose course is disgracing the profession. Say that those only who come up to the conditions laid down by the Association, and who comply in good faith with its requisitions, shall have a place, and you will encourage the well disposed schools to persevere in their efforts at improvement and reform, and ultimately break down those who pursue a contrary course. He said that the Committee of the National Medical Association, to whom this subject had been referred, proposed to allow one representative in the Association to all Colleges which had six professors, which gave an annual course of at least sixteen weeks, and which exacted from candidates for graduation, that they should be twenty-one years of age, have studied for three years, two of which must have been with some respectable practitioner, and should have attended two full courses of lectures. He was strongly of opinion, that if we desired to see the schools come up to this standard, we should not shut them out of our ranks. If we continued to receive representatives from them, he believed that we should ultimately destroy all who refused to comply with our demands.

Dr. H. S. Patterson thought that the thanks of the Society were due to Dr. Hays, for presenting the question in a nut-shell. The question was now clearly, whether the members of the medical schools were to sit in the National Association as *individuals* or as *professors.* As regards the feeling of the schools on this point, he was pretty certain that they did not ask the privilege as a body, and that very many faculties were not at all strenuous about retaining it. Be this as it may, however, we have the question now directly brought to an issue, by the Montgomery County resolutions on the one side, and Dr. Hays' amendment on the other. Dr. Hays' argument, that we should keep the schools in the Association to have them under control, Dr. Patterson said, appeared to him like the idea of "marrying a man to get rid of him." He really could see no force in it. But he could see much force in the objections urged against the inequality and injustice of the privileges claimed for the schools. To allow a faculty of six professors one representative—for they could not go below one—was certainly to establish a very great disproportion between professors and members of the county medical societies. And, besides this, there was another injustice; the professors were twice represented, once from the faculties, and again from the societies. Now, there was a very general feeling, which, whether just or not, it was wise to respect, that this distinction ought not to be made between professors and other members of the profession. It savored of an oligarchy; it made a sort of upper house, although sitting in the same body. It were far better for the gentlemen of the schools to come down to the same level with their brethren, and to consent to make the National Association a purely representative body. He was for adopting an equal ratio—not so high as to exclude sparsely settled districts, but, on the other hand, not low enough to make the Association an indiscriminate crowd. And he thought there would always be a fair proportion of professors in the body, as in fact they often went there now, from this city, as representatives of the College of Physicians, and of the County Medical Society. There need be no fear, he thought, that the Association would lose a control over the schools, because they were not to be allowed to knock at their doors with the privileges of close bodies. They would always be within the reach of a sound and healthy public opinion; and this they would never defy. At all events, he had little faith in the efficacy of mere legislation. And he was satisfied that the establishment of an inquisitorial censorship over the Colleges, such as Dr. Hays' plan seemed to imply, was exceedingly difficult. It would certainly lead to a delicate and dangerous assumption of power, which would be always most reluctantly exercised, from an indisposition to establish personal enmities.

Dr. Hays reiterated his views.

Dr. I. Parrish expressed his cordial approval of Dr. Patterson's remarks.

Dr. Condie was in favor of the amendment of Dr. Hays. His own opinions, which he had long since expressed, were in accordance with the tenor of the Montgomery county resolutions; but he knew that the sentiments which prevailed in the National Association were the other way, and he thought it inexpedient to tie the hands of the representatives whom we sent there, lest our vote should be lost. It was best for the present to be content with a *restriction* of college representation, and not to endanger the whole plan for improving the representation of the Association, in the attempt to accomplish too much.

The question was now taken on the amendment of Dr. Hays, and it was lost—yeas 20, nays 25. The first resolution of the series was then adopted.

The second resolution coming up, Dr. Stillé urged, that there was a palpable impropriety in the use of the language, "that the admission of delegates from colleges, &c., produces dissatisfaction among the mass of the profession;" as, on the contrary, the feeling at the late meeting of the National Association was nearly unanimous in favor of retaining a restricted representation of the colleges.

Dr. Wood said, that although his position prevented him from taking part in the discussion on the main question, he must be permitted to observe that the close vote just taken here, (20 to 25,) made it pretty obvious that the feelings of the great mass of the profession were less earnest on the subject than the resolution claimed.

After some further discussion, the second resolution was laid on the table, and Dr. Wimley subsequently withdrew it.

Dr. Hays hoped the third resolution would be also laid on the table.

Dr. Parrish urged its passage, deeming it strictly in conformity with the first resolution. It was adopted without a division.

Dr. Stillé moved to lay the fourth resolution on the table. This motion was lost, and the resolution was then adopted.

The preamble was then adopted.

Dr. Emerson offered a series of resolutions which were adopted unanimously, urging it, as an imperative duty, on all regular physicians in the State, to connect themselves with the county societies now organized, or to organize them in counties where they do not exist; and the county societies are requested to have these resolutions published in the newspapers of their localities.

Dr. Emerson made a report from the special committee appointed at the last annual session, to investigate the conclusions put forth by Drs. Gregory, of London, and Cazenave, of Paris, in relation to variola and vaccination. These conclusions are not sustained by the committee.

Dr. Emerson, from the Committee appointed at the last session, to memorialize the Legislature for the passage of an act to secure the gratuitous vaccination of the poor throughout the commonwealth, reported that the memorial had been printed and distributed. The committee was continued.

On motion of Dr. Corson, the committee on Publication was instructed to distribute the 150 copies of the Proceedings of the last session, remaining undisposed of, to prominent physicians of counties which have not yet organized county medical societies throughout the State, and to urge on them the immediate organization of county societies.

On motion of Dr. Fox, the resolution adopted at the first meeting of the present session, appropriating all the funds of the Society to the Committee on Publication, was reconsidered, and afterwards amended, by inserting the words, "after defraying the necessary expenses of the Society."

Dr. Gries asked and obtained permission to withdraw the Berks county report on spurious drugs.

Dr. Corson, in behalf of the Montgomery County Medical Society, offered a resolution inquiring of the State Society the legitimate construction of sec. 3d, art. 6th of the constitution: "Whether the said section applies to any others save those who actually compound, prepare and sell secret or patent medicines; or whether it applies equally to wholesale and retail druggists, or those who sell them, as one of the unavoidable results incident to the keeping of a drug store." Referred to a special committee, consisting of Drs. H. S. Patterson, Mayburry, and Wallace.

Dr. Hays, from the Committee on Nominations, made a report, which was objected to by Dr. Fox, on the ground that one of the persons nominated was constitutionally ineligible, not being a delegate. The report was referred back to the Committee.

Dr. Smith, under instructions from the Delaware County Medical Society, submitted a report on the subject of gratuitous attendance on the poor. At the suggestion of Dr. Condie, it was laid on the table till the afternoon.

The President announced Drs. Condie, I. Parrish, and Norris, as members of the Committee on Publication in addition to the ex-officio members.

The Society adjourned to four o'clock, P. M.

AFTERNOON SESSION.

The Society met, pursuant to adjournment, at four o'clock, P. M., the President in the Chair.

On motion of Dr. Condie, the Society took up the Report of the Delaware County Society on the subject of gratuitous attendance, when a report was read, which had been presented to the Philadelphia County Society on the same subject. This report expressed an opinion adverse to any action on the subject, which contravened the established usages of the profession, as laid down in the Code of Ethics. While not disposed, however, to narrow the limits within which the benevolence of the profession had been extended, the committee laid it down as a principle that medical attendance on paupers should be provided for at the expense of the community in which they lived. Almshouses should certainly be expected to compensate their medical officers; while, however, in hospitals supported by private charity and by funds bequeathed by charitable individuals, the services of physicians might with propriety be rendered gratuitously, the reputation accruing therefrom being considered a sufficient remuneration.

Dr. Smith would not have it supposed that there was a single word in the Philadelphia County report to which any member of the Delaware County Society took exception. But he thought the state of things in the country, on the subject of what was termed out-door attendance on paupers—whatever might be the rule in Philadelphia—was such as to call for the appointment of a committee to take the subject into consideration.

A brief debate ensued, in which Drs. Perkins, Corson, Emerson, Henry, Hays, and Condie participated; and, finally, on motion of Dr. CONDIE, the reports from both counties were referred to the Committee on Publication.

Dr. HAYS called up his resolution for the appointment of a committee to devise a plan for the arrangement of committees to report on diseases in connection with geological formations. It was adopted, and Drs. J. P. Hiester, Halberstadt, and Corson were appointed the committee.

Dr. H. S. PATTERSON, from the Committee to whom the queries of the Montgomery County Society had been referred, reported: That the Montgomery County Society be informed, that no one, who is in any way connected with the manufacture or sale of patent medicines, can be allowed to become or remain a member of State or County Society.

Dr. HAYS, from the Committee on Nominations, reported the following list of Officers for the ensuing year, and, on motion, the persons nominated were unanimously elected.

*President.*—HIRAM CORSON, M. D., of Montgomery.

*Vice-Presidents.*—GEORGE HALBERSTADT, M. D., of Schuylkill, JOHN T. RAY, M. D., of Mercer, J. D. ROSS, M. D., of Blair, C. F. PERKINS, M. D., of Erie.

*Recording Secretaries.*—HENRY S. PATTERSON, M. D., of Philadelphia, JOSEPH GIBBONS, M. D., of Lancaster.

*Corresponding Secretary.*—ISAAC HAYS, M. D., of Philadelphia.

*Treasurer.*—FRANCIS WEST, M. D., of Philadelphia.

*Censors.*—1st and 2d districts, G. B. WOOD, M. D., and G. W. NORRIS, M. D., of Philadelphia, WILMER WORTHINGTON, M. D., of Chester, JESSE YOUNG, M. D., of Delaware, J. W. GLONINGER, M. D., of Lebanon. 3d and 4th districts, J. M. DORSEY, M. D., of Huntingdon, J. H. CASE, M. D., of Perry, WM. MCILVAINE, M. D., of York, JOS. HENDERSON, M. D., of Mifflin, THOS. WOOD, M. D., of Lycoming. 5th and 6th districts, C. F. PERKINS, M. D., of Erie, W. McK. MORGAN, M. D., J. P. GAZZAM, M. D., J. DICKSON, M. D., and W. ANDERSON, M. D., of Alleghany.

*Delegates to the American Medical Association.*—C. INNES, M. D., of Northampton, ISAAC THOMAS, M. D., of Chester, F. S. BURROWES, M. D., of Lancaster, JOS. CARPENTER, M. D., of Schuylkill, THOS. VAN VALZEH, M. D., of Mifflin, R. E. JAMES, M. D., of Northampton, JAS. R. MCCOY, M. D., of Bucks.

On motion of Dr. EMERSON, it was resolved, that when the Society adjourns, it adjourn to meet in the city of Philadelphia, on the fourth Wednesday in May next.

On motion of Dr. CONDIE, the Delegates to the American Medical Association were authorized to appoint substitutes, in case of their inability to attend.

Dr. INNES now vacated the chair, and, in retiring from its duties, he expressed his acknowledgements to the Association, for the kindness and courtesy, which had been extended to him.

Dr. CORSON, President elect, was conducted to the chair by Dr. INNES, and in a few neat remarks returned thanks for the honor conferred.

On motion of Dr. CONDIE, Dr. Corson was requested to furnish an abstract of his address on taking the chair, for record in the minutes.

Votes of thanks were passed to the officers, and to the Philadelphia county delegation, and the Society adjourned sine die.

[The whole number of delegates in attendance, who signed the roll, was sixty-seven.]

---

THE VOLTAIC PILE.—The President of the French Republic has published a decree offering a reward of 50,000 francs (2,000*l.*) to such persons as shall render the voltaic pile applicable with economy to manufactures, as a source of heat, or to lighting, or chemistry, or mechanics, or to practical medicine. Persons of all nations may compete for this prize, and the competition is to be open for five years.

The Union Medicale states that in 1812 there were 537 medical men practising in Paris, whilst in 1851 there were 1,352, being an augmentation of 815 in 40 years. The population of Paris in 1812 was 547,756 inhabitants, or 1,018 persons for each medical man; at present it is 900,000, or 666 for each medical practitioner.

NOVEL TREATMENT OF ANEURISM.—We have been much interested during the last few weeks in watching the progress of a case of aneurism of the subclavian artery under the care of Mr. Fergusson, in which a novel and ingenious method of treatment has been adopted. In imitation of an occurrence which occasionally happens by accident in cases of aneurism, viz., displacement of the mass of fibrine, or a portion of it, which is usually present in such tumours, whereby, in consequence of alteration in the current of the blood, a spontaneous cure results, Mr. Fergusson has, by manipulation of the tumour, thrown loose a portion of the fibrine in the case alluded to, with the effect of instantaneously arresting all pulsation in the upper limb. In four days a feeble pulsation at the wrist could be detected, but the axillary has been pulseless since. The tumour itself, which was at first about the size of a small hen's-egg, has diminished considerably, and the throbbing within is now little greater than in the subclavian artery of the opposite side, while it has become more solid to the touch. To those familiar with the pathology and treatment of aneurism, and especially the fatal results which have hitherto followed all attempts at cure by operation on the subclavian on the tracheal side of the scaleni muscles, we need hardly point out the interesting character of the case now under Mr. Fergusson's care.—*Medical Times and Gazette, March* 6, 1852.

